# Exergaming for balance training of elderly: state of the art and future developments

**DOI:** 10.1186/1743-0003-10-101

**Published:** 2013-09-25

**Authors:** Mike van Diest, Claudine JC Lamoth, Jan Stegenga, Gijsbertus J Verkerke, Klaas Postema

**Affiliations:** 1 INCAS3, Dr. Nassaulaan 9, 9401 HJ Assen, The Netherlands; 2 Center for Human Movement Sciences, University of Groningen, University Medical Center Groningen, Hanzeplein 1, 9713 GZ Groningen, The Netherlands; 3 Center for Rehabilitation, University Medical Center Groningen, University of Groningen, Hanzeplein 1, 9713 GZ Groningen, The Netherlands; 4 Department of Biomechanical Engineering, University of Twente, P.O. Box 217, 7500 AE Enschede, The Netherlands

**Keywords:** Postural control, Sensors, Exergames, Balance training, Elderly, Fall prevention

## Abstract

Fall injuries are responsible for physical dysfunction, significant disability, and loss of independence among elderly. Poor postural control is one of the major risk factors for falling but can be trained in fall prevention programs. These however suffer from low therapy adherence, particularly if prevention is the goal. To provide a fun and motivating training environment for elderly, exercise games, or exergames, have been studied as balance training tools in the past years. The present paper reviews the effects of exergame training programs on postural control of elderly reported so far. Additionally we aim to provide an in-depth discussion of technologies and outcome measures utilized in exergame studies. Thirteen papers were included in the analysis. Most of the reviewed studies reported positive results with respect to improvements in balance ability after a training period, yet few reached significant levels. Outcome measures for quantification of postural control are under continuous dispute and no gold standard is present. Clinical measures used in the studies reviewed are well validated yet only give a global indication of balance ability. Instrumented measures were unable to detect small changes in balance ability as they are mainly based on calculating summary statistics, thereby ignoring the time-varying structure of the signals. Both methods only allow for measuring balance after the exergame intervention program. Current developments in sensor technology allow for accurate registration of movements and rapid analysis of signals. We propose to quantify the time-varying structure of postural control during gameplay using low-cost sensor systems. Continuous monitoring of balance ability leaves the user unaware of the measurements and allows for generating user-specific exergame training programs and feedback, both during one game and in timeframes of weeks or months. This approach is unique and unlocks the as of yet untapped potential of exergames as balance training tools for community dwelling elderly.

## Introduction

More than one third of the community-dwelling individuals aged 65 and older falls at least once per year [1,2]. Approximately 5-20% of the falls have serious consequences including major head trauma, major lacerations, or fracture and may lead to immobility or death [1,3]. The severity of the consequences of falls increases with ageing and the number of injuries and injurious falls strongly predict placement in a nursing home [4,5].

Impaired postural control with muscle weakness is an important predictor of falls within the elderly population [6,7]. Postural control is defined in this review as the ability to maintain, achieve, or restore a state of balance during any posture or activity [8]. Ageing has a detrimental effect on postural control either due to a specific pathology affecting a particular component of the sensory, motor and central processing systems, and/or as a consequence of a more general age-related deterioration of sensory and neuromuscular control mechanisms [9,10]. Appropriate control of posture underlies many motor skills and is an absolute pre-requisite for activities of daily living. Postural control entails accurately timed vestibular, visual, proprioceptive and somatosensory inputs for adaptive strategies for orientation and balance [9]. For the simple act of reaching for a cup, one must permanently monitor all the afore-listed inputs and perform subtle adjustments to coordinate movement. Integration of sensory inputs occurs without conscious attention, so people are routinely able to perform two tasks simultaneously, like talking and walking [11]. However, it has been shown that the control of posture, even in young adults, can suffer from the concurrent execution of dual tasks, thus placing a heavy load on working memory capacity [12,13]. The age-related loss of visual, proprioceptive, and vestibular sensitivity demands more attention for maintaining postural stability during standing and walking [14-16]. In that case, responses to dual tasking could destabilize motor activity [17,18]. Moreover, ageing is not only associated with impaired movement dynamics but also with a deterioration of cognitive processes involving working memory. One may thus pinpoint two major reasons for a reduced postural control during execution of a concurrent task: A greater need for conscious attention to maintain good postural control due to impaired sensory and motor system function and, by the same token, a reduced attentional and working memory capacity.

Many interventions for fall risk and fall rate reduction have been studied in the past years. Components of intervention programs include strength, balance and gait training, improving ambulation with improved footwear, walking aids, fall training, medication review, vision tests, fall sensors, home risk assessment and hip protectors [19,20]. Key components of fall prevention training programs for community dwelling elderly include balance, muscle-strength, flexibility and endurance [21]. The greatest effects on fall rate were seen in programs that included a combination of a high dose of exercise (>50 hours over the trial period) and challenging balance exercises [22].

A drawback of conventional exercise programs is however, that they suffer from low adherence, particularly if prevention is the goal as those programs usually start after one or more falls with serious consequences [22-24]. Exergames (exercise + gaming) appear promising for home-based balance and strength training for healthy elderly. Exergaming devices have several advantages compared to conventional exercises; exergaming can motivate people to practice and by performing dual tasks users can train both cognitive and motor skills. Additionally the focus of attention is not on the movements itself, but on the outcome of the movements in the game. This is important as in daily life one is also focused on the outcome of movements rather than consciously aiming to maintain balance [24-26].

Gaming technologies have become increasingly affordable and accessible over the past few years and exergames have therefore gained much interest in the field of mobility training for healthy individuals as well as for specific pathologies including stroke, SCI and cerebral palsy. Plow et al. reported in a scoping review of exergaming for adults with systemic disabling conditions that most of the studies reviewed showed potential to improve functional ability in the target population but that the field is still in its infancy and that there are few controlled trials [27]. The current review aims to provide an overview of the exergames that have been used for the specific purpose of training balance in the elderly population. Commercial systems developed specifically for training balance in elderly are rare [28] and most studies rely on off-the-shelf systems with commercial software not specifically developed for elderly or custom software developed for experimental balance training purposes. Although a rapidly growing number of studies are published about exergames, well-controlled studies are still rare. Therefore the present paper will not provide a systematic review of the literature, but provide an in-depth description and discussion of results obtained so far from existing commercial-of-the-shelf systems and custom-designed exergames to train balance. More specifically, 1) technologies and 2) outcome measures used and 3) the effects of the exergame interventions in the elderly population will be discussed. Finally, based on these results we will provide a conceptual framework of the possibilities of exergames as a balance training tool for elderly.

### Search criteria

Pubmed and Web of Science were searched to provide an overview of the technologies, outcome measures used, and the effects of exergames on balance ability. The keywords used in the search were: [serious games OR exergames OR virtual reality OR computer assisted OR video] AND [balance OR postural stability OR postural control] AND [training OR exercise OR fall prevention]. A selection was made based on the following inclusion criteria: The study should evaluate a commercial of-the-shelf system or evaluate or propose a custom exergame or VR-tool with game elements used for training balance in elderly or adults. Additional studies were identified by scanning reference lists. It was decided to exclude the studies involving pathological states including Cerebral Palsy (CP), Stroke, Acquired brain injury (ABI) and spinal cord injury (SCI) as these users show little similarity with community dwelling elderly, thereby including 13 studies.

### Exergaming devices

Exergame devices are controlled using a broad variety of sensor systems and, depending on the source of input, different algorithms are needed for game control and feedback. The most widely used sensors in exergame input devices include accelerometers, gyroscopes, infrared (IR) and RGB optical sensors/cameras and pressure sensors [29-34].

#### Inertial sensors

Inertial sensors encompassing accelerometers and gyroscopes are positioning sensors that measure accelerations and angular velocity respectively. A well-known commercial off-the-shelf game system that uses inertial sensors is the Nintendo Wii (Nintendo, Kyoto, Japan), which was introduced by Nintendo in 2006. Wii gameplay is controlled by the players’ movements, measured with a wireless hand-held controller, a Wii remote, in which a three-axis accelerometer and a single- and dual-axis gyroscope are embedded. By fusing the sensor data from the gyroscopes and the accelerometers, the Wii Remote can measure changes in direction, speed and acceleration with a sensitivity of ±1% [35]. Inertial sensors measure specific force and angular rate without an external reference. Consequently, errors build up quickly over time and the precise position of the device is difficult to deduce from these signals. The Wii remote therefore has an additional optical sensor on the controller that measures the position of a sensor bar, mounted on the television, which emits 2 IR light signals. The distance between the lights and the relative angle between the IR lights provide information about the position of the controller. Using these sensors the Wii controller is able to measure both rapid (using inertial sensing) and slow (using the optical sensor) movements.

Inertial sensors have also been used in wobble boards for training balance (see Table 1) [24,25,33]. These boards consist of an instable plate, which causes the user, while standing on the plate, to wobble, thereby controlling the game, for instance a ball in a maze [33], by shifting his weight in ML and AP directions. The movements are measured using a single orientation tracker (Xsens MTx Motion Tracker, Xsens Technologies, The Netherlands) which consists of three gyroscopes [36]. For global reference the MTx measures the direction of gravity and the magnetic north. The accuracy of the device is <1 deg, the update rate of calibrated sensor data: 512 MHz.

#### Pressure sensors

Pressure sensors are also used widely as a game input device [23,29-31,37-45]. For instance, the Wii Balance Board (WBB) consists of a board (51x31 cm) with 4 force transducers thereby allowing for calculation of the users center of pressure (COP) used for controlling games. Typical game tasks include shifting weight, taking poses or stepping on and off the WBB in the context of yoga or aerobics [42]. Comparable systems are a pressure mat [23] or panels with pressure sensors used for dancing games [30,31].

#### Camera systems

Inertial and pressure sensors hold the limitation that the user is in direct contact with a controller. Alternatively, camera systems provide the possibility to play games without holding or wearing input devices [38,40,46-48]. The Sony PlayStation Eyetoy (Sony, Park Ridge, New Jersey, USA) for instance, uses a color video camera with a software package that enables gesture recognition to play games. Games are controlled by the movements of the user itself, rather than via a controller that is held by the user. The Eyetoy has been used predominantly for (upper extremity) rehabilitation purposes [32,46-48]. A disadvantage of this system is that the camera system does not provide the accuracy necessary for playing faster games or taking high resolution measurements. Commercially available webcams are also being used to control exergames [40].

A recent development is the field of exergames is the XBOX 360 Kinect (Microsoft corp., Redmond, WA). Like the Eyetoy it uses gesture recognition rather than a hand-held game controller. The system captures depth and color information and generates a point cloud of colored dots. The software is able to calculate the 3D position of the dots, thereby creating a 3D image of the environment. Algorithms analyze the sensor data and calculate the position of the user’s body parts thereby allowing tracking of the users’ movements, even when a body part is occluded [49]. Like the Eyetoy the Kinect does not allow for measurements with a resolution and sample frequency comparable to high-end camera systems. Although several studies are being conducted, no publications relating to balance training have been published on the cutoff date for this paper.

### Outcome measures: internal and external

A broad variety of outcome measures for quantifying the effects of exergame interventions are reported in literature. Two different methods to assess performance can be discerned 1) during gameplay and 2) outside the game environment, in the present paper referred to as internal and external outcome parameters, respectively. Internal outcome measures are generated using an instrumented measurement tool and algorithms that convert the sensor data automatically into the outcome measure. The range of movement of the COP can for instance be measured using a pressure mat during a weight shifting game task [23]. External outcome measures are administered after gameplay. Examples include administering the Berg Balance Scale or sway variability during standstill after an intervention period [33]. Contrary to external outcome measures, internal outcome measures can provide the user with direct feedback during gameplay.

In several exergame studies the COP is used to control the game yet these measurements are not considered internal or external outcome measures as they do not quantify balance ability but only are used to play the game [38,39]. Table 1 provides an overview of outcome measures used in exergame studies, along with the technologies used to measure these parameters and the study results.

Clinical balance and mobility tests like the Berg Balance Scale (BBS) [50] and Timed Up and Go (TUG) [51,52] are abundantly used to quantify the effect of an exergame intervention on postural control in young and older adults [29,33,41,42,45,46] and are considered external measures. Other clinical tests that are used as outcome measures in the reviewed exergame studies include the Brunel balance assessment (BBA) [53], Anterior reach tests (ART) [54], Timed stair test (TST) [55], Stepping test (ST) [56], 1 minute walking test (1MWT) [57], 10 meter walking test (10MWT) [58], 30 seconds sit-to-stand test (30SST) [59], Community Balance and Mobility Scale (CB&M) [60], Star Excursion Balance Test (SEBT) [61], figure of eight-test [62], the tandem stance and one-leg stance [29,33,38,63]. All these tests are suggested to provide information about postural control during different standing and walking tasks.

In exergaming studies the use of external balance measures based on sensor data is slowly emerging, but the number of studies using instrumented quantification of postural control is still small [23-25,31,33,38,41-44]. Most of these studies assessed balance by quantifying sway variability in mediolateral (ML) and anteroposterior (AP) direction during quiet stance using forceplates, as is widely used to measure balance ability in young and older, healthy and pathologic individuals [38,41,43,44,64-67]. Postural control was also assessed using trunk acceleration time series where regularity, variability and smoothness of the trunk acceleration patterns in ML and AP direction were quantified using the root mean square, sample entropy and mean power frequency respectively [25,33]. Other instrumented external outcome parameters that are supposed to indirectly determine balance and were reported in the studies reviewed include measurement of the ankle dorsiflexion, Limits of Stability (LOS), quadriceps strength, tactile acuity and body weight [41] and dynamical postural stability index (DPSI) [24,68], the latter being a method in which the subject jumps and has to stand still for 3 seconds on a forceplate after landing.

For measuring game enjoyment, motivation, perceived exertion, cognitive abilities, mental health, self-efficacy and balance confidence, questionnaires including custom made questionnaires regarding game enjoyment [23] and intrinsic motivation [24], semi-structured interviews [40], the digit symbol substitution test (DSST) [69], the SF 36 mental component summary [70], Fall Efficacy Scale [71], Falls Efficacy Scale international (FES-I) [72] and Activity Specific Balance Confidence (ABC) scale [73] are used respectively.

Internal outcome measures used in the studies reviewed include step timing and the percentage of missed targets [31] and the total movement range of COP in ML and AP direction [23], all measured using pressure mats. Several exergame studies evaluated the effect of Wii games or custom games that make use of the Wii balance board, but few studies use this board as a source for internal outcome measures [42,43]. The validity of the internal instrumented measures used in the studies reviewed is under debate as they are often game-specific and although sometimes correlated with established measures, they are often not validated using follow-up studies on fall risk. An increase in these measures can reflect both an improvement in balance ability as well as a training effect of the game. Of the studies reviewed, only Smith et al. validated their internal outcome measures [74].

### Effects of exergaming on balance ability in adults and elderly

Of the 13 studies reviewed, 5 used a (randomized) controlled trial design [23,24,31,42,44] and 8 did not use a control group [25,30,33,38,40,41,43,75]. The results are summarized in Table 1. Two studies aimed to improve balance in healthy adults and used external outcome measures, both clinical and instrumented, for balance assessment [24,41]. Significant effects were found in SEBT score in posterolateral and posteromedial direction after playing wobble board games controlled using weight shifts. No effects were found using the instrumented measure, the DPSI [24]. Playing WiiFit games using a WBB significantly improved sway variability during unilateral stance for both limbs and lower limb strength but no effects were found using clinical measures including 6MWT and TUG [41]. Assessment of game enjoyment, motivation to exercise and game experience in healthy adults was assessed using questionnaires and interviews and it was found that all participants enjoyed playing the exergames [23,40].

**Figure 1:**
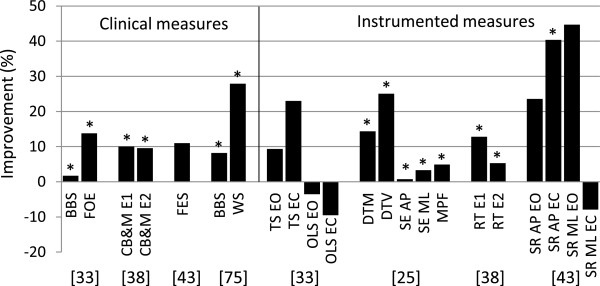
**Summary of results of uncontrolled studies evaluating training effect of exergames on balance ability of elderly.** The vertical axis represents the percentage improvement between pre- and post-intervention on the outcome measure provided on the horizontal axis. The bars on the left side of the solid vertical line indicate clinical measures, the bars on the right side instrumented measures. BBS = Berg Balance Scale, FOE = Figure of Eight, CB&M = Community Balance and Mobility scale, E1 = experimental group 1, E2 = experimental group 2, FES = Fall Efficacy Scale, WS = Walking speed, TS = tandem stance time, OLS = one-leg stance time (both TS and OLS are measured in seconds), DTM = Dot gaming Task Mean, DTV = Dot gaming Task Variability, SR = Sway Root mean square values, EO = eyes open, EC = eyes closed, SE = sample entropy, AP = antero-posterior direction, ML = medio-lateral direction, MPF = mean power frequency, RT = reaction time, SR = Sway Root mean square values, * indicates a significance of P < 0.05.

**Figure 2:**
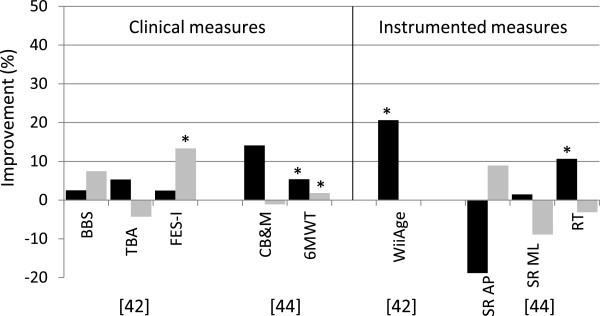
**Summary of results of controlled studies evaluating training effect of exergames on balance ability of elderly.** The vertical axis represents the percentage improvement between pre- and post-intervention on the outcome measure provided on the horizontal axis. Dark bars indicate the experimental group, light bars the control group. Note that [42] found an improvement in the control group where the experimental group did not improve. The bars on the left side of the solid vertical line indicate clinical measures, the bars on the right side instrumented measures. BBS = Berg Balance Scale, TBA = Tinetti Balance Assessment, FES-I = Fall Efficacy Scale International, CB&M = Community Balance and Mobility scale, 6MWT = 6 Minute Walk Test, SR = Sway Root mean square values, AP = antero-posterior direction, ML = medio-lateral direction, RT = reaction time. * indicates a significance of P < 0.05.

The effects of exergaming on age-related impaired postural control in elderly was examined in 9 studies [25,30,31,33,38,42-44,75]. One study used internal, 7 studies external and 1 study used both internal and external measures to assess balance ability. Of the studies using external measures, 3 used clinical, 1 used instrumented and 4 both clinical and instrumented measures. The largest effects were observed for clinical tests assessing balance during walking and during several standing tasks; significant improvements over the intervention period were found in CB&M score [38,44], narrow walk time, ABC scale [30], BBS [33,42,75], walking speed [75], 6MWT [44] and FOE [33]. Standing balance was assessed using summary statistics in 4 studies using various instrumented measures but few found significant improvements over the intervention period, except for the sway variability in AP direction in eyes closed condition [33,38,43,44]. Also reaction time improved significantly [38,44]. Internal balance measures were used by 2 studies; improvements in step timing and percentage missed targets were observed after five 4-minute trials. Additionally, it was found that young adults outperform older adults [31]. As is shown in Figures 1 and 2, older adults predominantly improved on measures with a dynamic component such as walking, reaching and performing standing tasks. The effects of exergame programs are not often found significant when instrumented parameters measuring postural sway during quiet stance are applied. Also significant differences between intervention and control groups are scarce. Of the 13 studies reviewed 10 reported improvements in at least one balance measure after an exergame intervention period. The tasks performed in these effective games included stepping and weight shifting tasks.

**Table 1:** Overview technologies and results of studies evaluating exergames for balance training in elderly and adults

	**Sample characteristics & Study type**	**Technologies used**	**Game elements and tasks**	**Intervention design**	**Internal outcome measures**	**External outcome measures**	**Results**
					**Feedback to user (FB)**		
Studenski [30]	E: Healthy elderly (N = 36) Age: 80.1 ± 5.4 Study type: UCT	Dance pad containing four panels	Dance game (DancetownTM) controlled by stepping movements in forward, backward left and right directions.	24 x 30 min sessions over 3 months. Total = 720 min.	Not used FB user: Game performance	- BMI - blood pressure - Pulse - SPPB - NWT - Digit symbol substitution test - SF36 PMC - ABC scale	SF 36 PMC, NWT and ABC scale improved (p < 0.05) No significant effects on other outcome measures
Smith [31]	E 1: Healthy elderly (N = 26) Age: 78.9 ± NR E 2 and E 3: Healthy elderly (N = 20) Age: 79.6 ± NR C: Young adults (N = 20) Age: 28.4 ± NR Study type: CT	Dance mat containing four panels	E 1: Stepping to the left, right, left forward or right forward on panels corresponding to arrows shown on a display. Arrow drift speed varied. E 2: Same game with constant arrow speed. E 3: Manipulated arrow speed and appearance	E 1: 4 min E 2 and E 3: 5 x 4 min trials < 1 hr. Total Studies 2 and 3 = 80 min.	-Step timing -% missed targets FB user: not reported	Not used	Step timing improved (p < 0.05) after multiple trials & group effect; young outperformed elderly (p < 0.05). % missed targets decreased after multiple trials (p < 0.05) & group effect; young adults outperformed elderly (p < 0.05).
Kosse [33]^1^	E: Healthy elderly (N = 9) Age: 77 ± 5.0 Study type: ITS	Wobble board	Moving a ball through a maze using weight shifts without lifting the feet	18 x 20 min. over 6 weeks Total =360 min.	Not used FB user: Game performance	- BBS - FOE - TS EO and EC - OLS EO and EC	BBS and FOE improved (p < 0.01) TS and OLS EC & EO did not improve
Fitzgerald [24]	E: Healthy adults (N = 11) Age: 25.4 ± 2.1 C: Healthy adults (N = 11) Age: 26.9 ± 3.2 Study type: RCT	Wobble board	E: Controlling a ball using weight shifts without lifting the feet C: Postural stability training on a wobble board.	3 x 15 min. p/wk for 4 weeks Total = 180 min.	Not used FB user: Game performance	- DPSI - SEBT - Intrinsic motivation inventory	DPSI and SEBT: no group effects. Intrinsic motivation: higher (p < 0.01) score on interest/enjoyment category
Lamoth [25]^1^	E: Healthy elderly (N = 9) Age: 77 ± 5.0 Study type: ITS	Wobble board	Moving a ball through a maze using weight shifts without lifting feet.	18 x 20 min over 6 weeks Total = 360 min.	Not used FB user: Game performance	- DT - Standing FP& TS: trunk acceleration patterns in AP and ML: variability, regularity, smoothness.	Performance on DT improved (p < 0.05), Postural control indexed by variability, regularity and smoothness improved (P < 0.05)
Betker [23]	Healthy Adults (N = 8) Neurological patients (N = 7) Age: 15 – 72 Study type: CT	Pressure mat, 53 × 53 cm, 256 pressure sensors.	Weight shifts in AP and ML direction with cognitive tasks.	3 × 10 min. Total = 30 min.	During gameplay: game performance, COP position After gameplay total ROM ML & AP directions.	- Questionnaire: 9 questions about enjoyment, motivation to exercise, game difficulty	Internal outcome measures: NR Questionnaire: Games are challenging, attractive and more appealing than traditional exercises.
Bisson [38]	E 1: Healthy elderly (N = 12) Age: 74.4 ± 3.65 E 2: Healthy elderly (N = 12) Age: 74.4 ± 4.92 Study type: ITS	Camera tracks red gloves, player projected in Virtual Environment. Force platform measures COP.	E 1: Moving cursor using weight shifts E 2: Catch 'Falling’ balls by reaching the arms without lifting feet	2 x 30 min. p/wk for 10 weeks Total = 600 minutes	Not used FB user: Game performance	- Sway variability AP and ML - CB&M - RT	Sway variability: no significant group effect. CB&M and RT: both groups improved (P < 0.05)
Lange [40]	E: Healthy adults (N = 7) Age: 16-43 Study type: UCT	Webcams, LED markers, custom step-based game.	Dance and step-based exercises in forward, backward, left, right and diagonal directions	Not specified	Not used FB user: Not reported	- Semi-structured interviews about game experience	All participants reported enjoying the experience
Nitz [41]	E: Healthy adults (N = 8) Age: 46.6 ± 9.9 Study type: UCT	Wii balance board	WiiFit: Yoga, balance, aerobic and strength pre-programmed activities.	2 x 30 min p/wk for 10 weeks. Total = 600 min.	Not used FB user: Game performance	- 6MWT - TUG - TUGcognitive - Step test - BS (foam, EC) - OLS (EO) - LOS - RT - Ankle dorsiflexion - LLS - Body weight	OLS for both limbs and lower limb strength improved (P < 0.05) Other outcome measures: no significant improvement.
Williams [42]	E: Elderly with increased fall risk (N = 15) Age: 76.8 ± 5.2 C: Elderly with increased fall risk (N = 6) Age: 76.5 ± 4.8 Study type: CT	Nintendo Wii, Wii balance board, walking frame.	E: WiiFit, balance and aerobic exercises controlled using stepping on the board, shifting weight and performing poses. C: Standard care exercise/education programme	E: 2 game sessions per week for 12 weeks. Total = Not reported C: 12 week exercise/education programme	Wii Age; number calculated by the Nintendo Wii based on game results. FB user: Game performance, Wii Age.	- BBS - TBA - FES-I	No group effect on BBS and TBA. Control group improved on FES-I (P < 0.05). Game group improved on Wii-age (P < 0.05)
Young [43]	E: Healthy elderly (N = 6) Age: 84.1 ± 5.1 Study type: ITS	Wii balance board, custom games.	Catching apples and popping balloons using COP shifts without lifting feet.	10 x 20 min. over 4 weeks Total = 200 min.	Not used FB user: Real-time visual FB of current COP position	- Sway variability AP EO & EC - Sway variability ML EO & EC - FES	Sway variability AP EC improved (p < 0 .05) Other sway measures and FES showed trend towards improvement.
Heiden [44]	E: Healthy elderly (N = 9) C: Healthy elderly (N = 6) Age : 77 ± NR Study type: CT	2 Force plates (25x10x1.5 cm)	E: Playing tennis game 'Pong’ controlled using weight shifts in AP and ML directions and a dynamic stepping routine + chair exercise program. C: Chair exercise program.	E: 2x (16 x 60 min. chair exercises + 30 min. exergaming) p/wk for 8 weeks. Total exercise = 1440 min. and gaming = 480 min. C: 16 x 60 min. over 8 weeks Total = 960 min	Not used FB user: Real time visual FB of COP movement	- Postural sway AP - Postural sway ML - RT dual task - CB&M - 6MWT	RT during dual task and CB&M improved (p < 0.05) in intervention group Both groups improved on the 6MWT (p < 0.05).
Agmon [75]	E: Balance impaired elderly (N = 7) Age: 84 ± 5 Study type: ITS	Nintendo Wii, Wii balance board	4 balance games controlled using weights shifts and stepping on the balance board	3x30 min p/w for 3 months. Total = 1170 min	Not used FB user: Game performance	- BBS - Timed 4-meter walk test - PACES	BBS and walking speed improved (P < 0.05). No effect on PACES.

E = Experimental group; C = Control group; N = Number of participants; RCT = Randomized controlled trial; UCT = Uncontrolled trial; CT = Controlled trial; ITS = Interrupted time series design. When no p values are presented in the table, they were not provided in the paper, ^1^=Same data set; NR = Not reported; ROM = Range of movement; ML = Medio-lateral; AP = Anterior-posterior; COP = Centre of pressure; SF36 PMC = SF 36 physical & mental component; NWT = Narrow walk time; DPSI = Dynamic postural stability index; SEBT = Star excursion balance test; FOE = Figure of eight; EO = Eyes open; EC = Eyes closed; DT = Dot task; RT = Reaction time; LLS = Lower limb strength; TUG = Timed-up-and-go; 6MWT = 6 minute walk test; LOS = Limits of stability; BMI = Body mass index; SPPB = Short physical performance battery; FES-I = Falls efficacy scale international; ABC scale = Activities specific balance confidence scale; BBS = Berg balance scale; TBA = Tinetti balance assessment; FES = Falls efficacy scale; CB&MS = Community balance and mobility scale; FP = Feet parallel; TS = Tandem stance; OLS = One-leg stance; BS = Bilateral stance; PACES = Physical activity enjoyment scale.

## Discussion

The present paper aims to provide a description and discussion of technologies and outcome measures used in exergames for balance training, covering both commercial off-the-shelf and custom hardware and games.

Recent developments in the field of sensor technology unlock great opportunities for using exergames as balance training tools. The studies reviewed applied inertial, pressure, and optical sensors for controlling exergames. Inertial and pressure sensors were used mostly [24,25,30,33,41-44], but camera systems are also becoming more widely used [38,40,46]. Cameras and gesture recognition software are expected to increase in popularity for rehabilitation purposes as they are getting cheaper, accuracy is increasing and gesture recognition is improving; these are important conditions for use in the home environment [76]. Inertial sensors however also become smaller and more energy efficient, thereby making them less obtrusive during gameplay [77]. Developments in the field of energy scavenging might even make manual recharging of batteries of inertial sensors obsolete [78]. Additionally the developments in the field of wireless sensor networks enable faster transfer and fusion of data of multiple inertial sensors, thereby enabling high sample frequency and more accurate measurements.

The broad variety of sensors, outcome measures and study designs used in the exergame studies reviewed indicates that the field of balance training is young and rapidly developing. Of the 13 studies included, 5 used a controlled trial design [23,24,31,42,44]. Of the studies that used an interrupted time series design, few included adequate number of participants and/or a control group. Moreover, studies are hard to compare as they use a broad variety of balance outcome measures, making it incongruous to draw conclusions regarding the effects of interventions.

In this paper internal and external outcome parameters were discerned and external parameters were classified as either clinical or instrumented. Significant improvements in balance ability were observed in studies assessing balance during walking or performing tasks using clinical tests [24,30,33,38,42,44,75]. The advantage of clinical balance tests is they are validated and easy to administer. A disadvantage however is that most of them only provide a global assessment of balance performance, and suffer from limitations including ceiling effects and limited precision to detect small changes in balance ability, which makes it onerous to estimate clinical relevance [50,52,60,72,79]. For instance, if a Berg Balance Score is higher or walking speed is faster, the cause of the change remains unclear. The exact aspects of postural control that are influenced by the intervention thus remain unclear.

Instrumented outcome measures can provide detailed information about changes in postural control. Only a limited number of the studies reviewed used objective instrumented outcome measures, and the measures applied in these studies used mainly summary statistics like the RMS of the amplitude of COP recordings. Many of these measures appeared unable to detect small changes in postural control, some of which actually were observed using clinical measures, as showed in Figure 1, Figure 2 and Table 1[24,33,38,43,44]. Using summary statistics may mask variability in postural sway patterns. There is a growing recognition that the time-varying structure of postural sway patterns contains valuable information on healthy and pathologic motor control [18,80-84]. To reveal these sway patterns, different types of data analysis techniques are necessary. A time-dependent analysis of variability and stability using measures derived from the theory of stochastic dynamics is a relatively new approach to quantify postural variability and stability [18,80-84]. Nowadays a wide variety of methods for quantifying balance ability using sway patterns has been applied and shown sensitive to detect change due to aging and pathologies including stabilogram diffusion plots (SDP) [81], recurrence quantification analysis (RQA) [85], detrended fluctuation analysis (DFA) [83,86], sample entropy (SEn) [87], Lyapunov exponent (λmax) [88] and Rambling and trembling analysis [89]. The new methods assess fluctuations in postural control signals and are suggested to be associated with the neuromuscular mechanisms underlying postural control [81]. These measures, based on the time-varying structure of postural sway patterns, are not depending on a specific sensor, but can be applied on various data sources including COP and acceleration data. Unlike clinical measures no healthcare professional is needed to guide the measurement and no ceiling effects are observed. However, the optimal selection of discriminative sway parameters is under continuous dispute; no gold standard is present for the quantification of postural control. In addition to the information provided about postural control, key aspects that remain to be examined in this respect are the accuracy, sensitivity and reliability of outcome measures. Internal measures hold the advantage that they are based on data recorded over a timeframe of hours, days or even weeks, since data is acquired constantly during gameplay, whereas external measures rely on short balance tests independent from the game environment. Internal measures on the other hand are calculated using sensor systems that need to be affordable for the individual user, thus compromising on accuracy of equipment is inevitable and the quality of measurements is usually lower than lab equipment used for external measures. However, as high quality sensors rapidly become smaller and more affordable, internal outcome measures can be based on data recorded with high precision and the challenge will focus on finding outcome measures that are reliable and sensitive to change in balance control of community dwelling elderly playing exergames.

Recent developments in sensor technology, game design and cheap processing power offers the unique possibility to calculate the above mentioned type of instrumented outcome measures during training activity. This clears the road for several breakthroughs in balance assessment and training. First, balance can be measured while playing exergames, leaving the user unaware of the measurements with no involvement of a healthcare professional or need for measurements in a laboratory setting. Second, the measurements allow for analysis over a variety of timeframes; balance can be quantified in the order of minutes and can serve as direct input in gameplay but results can also be stored and compared with measurements over months or even years. This allows personalized training programs and feedback which has shown to increase learning rate [90]. Moreover, measurements can be compared with peers using telemonitoring principles.

An additional advantage of exergames is motivation, although it is hardly examined quantitatively and compared with control treatment. The reviewed studies showed that exergame intervention groups found the training more appealing than traditional exercises [23], were more motivated to exercise [24] and showed better improvements than controls on clinical outcome measures [29,34,40,42].

We propose to utilize the latest advances in sensor technology and develop algorithms to analyze multivariate time series of postural control over different time-scales, for assessment of postural control both during gameplay as well as after gameplay, so to derive specific information regarding the postural control of the user and thereby adapting the exergame training program most efficiently to the individual user. Future developments should focus on developing algorithms that convert sensor data into information regarding postural control.

## Conclusion

Exergames have shown to hold interesting opportunities for improving balance ability in older adults. Although the number of controlled studies examining exergames remains small, the included studies report high enjoyment and motivation to perform exercises and several studies indeed showed an increase in balance ability using clinical and instrumented outcome measures after the training period. Current balance outcome measures however contain some caveats. We propose to utilize the recent advances in sensor technology and data analysis algorithms for quantification of balance ability during exergame training sessions, thereby unlocking new possibilities that exergames encompass for improving balance ability in community dwelling elderly.

## Competing interests

The authors declare that they have no competing interests.

## Authors’ contributions

MvD was involved in the conception of the review, the search and analysis and interpretation of the studies, and the writing of the manuscript. CJCL was involved in the conception of the review, interpretation and writing of the manuscript. JS, KP, and GJV were involved in revising of the manuscript. All authors read and approved the final manuscript.
